# Comparative Analysis of Healthy Lifestyles in Peruvian University Students During and After the COVID-19 Pandemic

**DOI:** 10.1155/tswj/2664351

**Published:** 2025-01-30

**Authors:** Carme Y. Baltazar-Meza, María Custodio

**Affiliations:** ^1^Academic Department of Education, Faculty of Education, Universidad Nacional del Centro del Peru, Huancayo, Peru; ^2^Academic Department of Human Medicine, Faculty of Human Medicine, Universidad Nacional del Centro del Peru, Huancayo, Peru

**Keywords:** habits, health, lifestyles, university students

## Abstract

The COVID-19 pandemic has provoked a worldwide mental health crisis, affecting university students in an exacerbated way, exposing them to the risk of emotional isolation and development of eating disorders. The present study aimed to examine the quality of healthy lifestyle practices in university students from the Faculty of Human Medicine and the Faculty of Education of a Peruvian university during and after the COVID-19 pandemic. This study was conducted during the academic period 2020 and 2023. Data collection was performed between July and August 2020, during the first wave of the COVID-19 pandemic (M1) and between November and December 2023, post-pandemic (M2). In M1 and M2, 370 and 82 students participated, respectively. The results of the Mann–Whitney test revealed significant differences between the total healthy lifestyle score of students in M1 and M2. In the nutrition domain, there are significant differences between the scores obtained in M1 and M2, revealing a decrease in nutritional care. Significant differences were also found between both faculties in substance use in M1 and physical activity practice in M2. Although a trend toward the adoption of healthy habits was observed in the initial stages, the longitudinal analysis reveals a generalized decrease in concern for health and well-being. Medical students, on the other hand, showed greater resilience and more successful adaptation to new circumstances, evidencing the importance of academic training in the promotion of healthy lifestyles. These results underscore the need to implement intervention strategies aimed at promoting healthy habits in the student population, especially in contexts of health crisis.

## 1. Introduction

In late December 2019, an emerging infectious disease named COVID-19 was identified and became a global pandemic. Countries around the world implemented various mechanisms to curb such infection and mitigate it. Mandatory social isolation was one of them, with drastic effects in social and economic fields [1, 2], especially in the lifestyle of people around the world and in particular of university students [3]. Changes in dietary habits during the COVID-19 pandemic were complex. While an increase in the preparation of home-cooked meals was observed, which could suggest an improvement in nutritional quality, an increased consumption of ultra-processed foods was also detected [4]. These changes were influenced by various factors, such as the economic situation, food availability, and changes in the family environment. Consequently, the healthy lifestyles of college students will vary according to the adoption of healthy lifestyle habits such as physical activity, nutrition, social relations, and psycho-emotional and spiritual aspects, which, if properly maintained, guarantee the integral development of the person [5]; otherwise, they can cause serious health problems.

The pandemic has led to increased levels of stress among students, resulting in disruptions in the daily lives and eating patterns of university students worldwide. This trend has been most evident among women, and there has been an increase in the prevalence of eating disorders [6, 7]. World Health Organization (WHO) notes that the university student population, an important segment of the young adult population, faces significant lifestyle changes, generally as a result of alterations in their eating patterns, sleep, and the intensity of their studies [8, 9]. Previous studies have shown that college students frequently adopt unhealthy lifestyles, characterized by smoking, poor diet, sedentary lifestyle, and excessive alcohol consumption. These habits increase the risk of developing chronic diseases such as obesity, overweight, and mental disorders [10]. The pandemic has exacerbated this problem, further altering the eating and physical activity habits of college students [11]. Confinement, associated with increased consumption of ultra-processed foods and less physical activity, raised concerns about the risk of developing chronic diseases such as obesity, overweight, and mental disorders [12].

In today's world, disease prevention and health promotion are key priorities that are influenced by lifestyle factors such as sleep quality, diet, body weight, physical activity, and substance use. These behaviors are essential for reducing disease risk and improving overall health [13]. During the COVID-19 pandemic, college students showed increased physical and mental health disorders, poor nutrition, and decreased physical activity, which significantly affected their overall health and lifestyle [14]. Studies also highlight that significant lifestyle changes among students may persist beyond the pandemic [15, 16]. The United Nations in 2022 indicates that in Latin America and the Caribbean, 22.5% of people do not have sufficient means to access a healthy diet. In the Caribbean, this percentage increases to 52% of the population, in Mesoamerica, the figure is 27.8%, and in South America, it is 18.4%. While the region is a significant global food provider, it has been disproportionately affected by the pandemic. Rising rates of undernourishment, food insecurity, and obesity have been observed, which have been identified as risk factors for adverse COVID-19 outcomes [17].

Most of the studies conducted in Latin America and the Caribbean during the COVID-19 pandemic on lifestyles have been carried out in the general population and have concluded that lifestyle modifications during the contingency have had a negative impact on the health of the population [2, 18, 19]. A study conducted on college students during the pandemic revealed that they had unhealthy lifestyles and a moderate association between chronotype and unhealthy lifestyles revealing a risk situation in the future [8]. Another recent study shows that college students' health and well-being during the pandemic varied, and daily experiences were influenced by both individual characteristics and contextual factors, highlighting the need for targeted interventions [20]. In Peru, the COVID-19 pandemic arrived unexpectedly, and it was not possible to establish whether university students had managed to internalize the health care they should have, based on the lifestyles they practiced. The context of the pandemic led young people to spend more time at home, to be less active, and to have less social interaction, with detrimental consequences for their health and emotional well-being [21]. On the other hand, the Peruvian Ministry of Health has indicated that remaining passive most of the time leads to overweight and obesity problems. This organization recommends practicing at least four healthy habits such as physical activity, adequate nutrition, reduction of alcohol consumption, and avoidance of tobacco consumption [22]. Unfortunately, research also reveals that college students do not always make the best decisions when it comes to their health. The present study aimed to examine the quality of healthy lifestyle practices in university students of the Faculty of Human Medicine and the Faculty of Education of a Peruvian university during and after the COVID-19 pandemic.

## 2. Materials and Methods

### 2.1. Design and Selection of Participants

A descriptive study with a longitudinal survey–based design was conducted. This study was conducted between July and August 2020, during the first wave of the COVID-19 pandemic (M1) and between November and December 2023, when the COVID-19 wave (M2) had already passed. This study was carried out in the university population belonging to the faculties of Human Medicine and Education of the National University of Central Peru during the academic period 2020 and 2023. The sample size was not estimated, since the survey was sent to the entire target population to collect data from all students (participants), since the aim was not to collect data from a sample but from the entire population. The inclusion criteria were all students of the faculties in question.

### 2.2. Data Collection

Given the health restrictions during the COVID-19 pandemic, the online questionnaire was considered the best option for data collection. During the first wave of the COVID-19 pandemic, 370 students participated in the survey, of which 315 were female (85.1%). In the second wave, 82 students participated, of which 63 were female (76.8%). The survey was collected through Google online forms, and for data collection, the snowball strategy [23] was used, facilitating participants to share the link to access the questionnaire with other students. At both moments, sociodemographic data of interest for the dimensions included in the study, such as age, gender, and semester, were collected.

### 2.3. Measuring Instrument

The instrument used was the survey “How is my lifestyle?” proposed by the Pan American Health Organization (PAHO) [24, 25]. The use of this instrument was considered because the acquired healthy lifestyle assessment scale demonstrates high reliability, with Cronbach's alpha values exceeding 0.67. The instrument is composed of 10 domains (45 items): (1) relationship with others, (2) physical activity, (3) rest, (4) nutrition, (5) oral health, (6) sexuality, (7) mobility, (8) substance use, (9) sense of life, and (10) environment. The score assigned to each item is ordinal, with 0 = never or almost never, 1 = sometimes, and 2 = always/almost always. The healthy lifestyle habits scoring scale indicates that a score < 40 qualifies as a high-risk lifestyle, 41–58 qualifies as a lifestyle with areas for improvement, 59–69 qualifies as an adequate lifestyle, and 70–80 qualifies as a healthy lifestyle.

### 2.4. Ethical Consideration

The study was approved by the Research Ethics Committee of the Universidad Nacional del Centro del Perú through CEI-UNCP-Nº055-2023 dated June 30, 2023. All participants were informed of the objective of the study and the confidentiality of the data collected through the informed consent form. It was also explained to them that their authorization could be withdrawn or canceled at any time.

### 2.5. Statistical Analysis

Statistical analysis of the data was performed using IBM SPSS Statistics software (Version 29.0; IBM Corp., 2023), using descriptive statistics to calculate the frequencies and percentages in the characterization of the population group. For the comparison of means between groups, the t student test was used when the variable consisted of two groups, as in the case of sex and faculty. In the case of variables with three or more groups, a one-factor ANOVA test was applied to determine differences between means. In addition, frequencies and percentages were calculated according to the ranges for the evaluation of the score obtained in healthy lifestyle. Frequency distribution was performed for each of the groups of the different variables considered in the study. Subsequently, the difference between the means of the two groups surveyed was evaluated. Previously, the Shapiro–Wilk test was used to determine the normality of the data. Comparison of the samples was performed with the nonparametric Mann–Whitney test. Data analysis was performed with a significance level (alpha) of 0.05.

## 3. Results

### 3.1. Sociodemographic Characteristics of the Participants

A lifestyle survey was applied to Peruvian university students in 2020 and 2023. In the 2020 population group (M1), of the 370 participants, 85.1% were women, mainly students of Education (84.3%) and Medicine (15.7%) between 18 and 24 years of age (94.6%). In the population group of 2023 (M2), the behavior was similar, predominantly female (76.8%), age range: 18–24 years (74.4%). In both population groups, female participation was concentrated in the tenth semester, in M1 (40.2%) and M2 (63.5%). The participation was 39.0% in Education and 61.0% in Medicine.

### 3.2. Significant Differences in Lifestyle

The results evidenced no significant differences in the total healthy living scores between males and females (*t* = −0.704, *p*=0.482), age (*F* = 1.089, *p*=0.325), and semester (*F* = 1.165, *p*=0.219) in M1. Similar behavior was observed in the areas of physical activity and nutrition among students from the Faculties of Education and Medicine (*t* = −1.066, *p*=0.287). However, as a function of substance use, a significant difference was found between the scores of the two faculties (*t* = 2.012, *p*=0.050). In M2, the results were like what was observed in M1, revealing no significant differences as a function of gender (*t* = −0.150, *p*=0.881), age (*F* = 0.976, *p*=0.522), and semester (*F* = 1.096, *p*=0.380). In the Physical Activity domain, significant differences were found between the scores obtained by participants from different faculties (*t* = 0.481, *p*=0.045). However, both M1 and M2 are in the very good range; with human medicine students showing the best healthy living habits.

### 3.3. Distribution of Healthy Lifestyle Scores

Table 1 shows the distribution of scores obtained categorized in each of the ranges for each variable in M1. Of the total participants in 2020, 46.49% were female and 7.03% were male. Most of the participants were in the score ranges from 59 to 69 and from 70 to 80, suggesting that in general the level of healthy lifestyle in the studied population is moderate to high. 50.27% of the participants were people between 18 and 24 years of age who were in the 70 to 80 healthy lifestyle range. The results also revealed that a large part of the participants (45.41%) belonged to the Faculty of Education, 10th semester (35.41%) who were in the range of 70–80.

In the same way, frequencies were calculated according to the ranges for the evaluation of the score obtained in healthy lifestyle for each group and variable (Table 2). Of the total number of participants in 2023, 24.39% were females who were in the range of 70–80 (their lifestyle keeps them healthy), while 8.52% were males who were in the same range. In addition, 24.39% of the participants were found to be in the range between 18 and 24 years old which corresponded to the 70 to 80 range of healthy living. The results also showed that the participants with the best healthy lifestyle in 2023 were the Human Medicine students (26.83%). Regarding the semester, it was found that tenth semester students presented the best healthy lifestyle range (14.63%). In both population groups, students who are about to finish their professional training are presented with a better lifestyle.

### 3.4. Comparison of Lifestyles Between Population Groups of Respondents in 2020 and 2023

Table 3 shows the Shapiro–Wilk normality test for the M1 and M2 population groups. The results reveal in most of the dimensions a *p* value lower than the significance level, except in M2 with respect to the healthy lifestyle dimension, where the *p* value was higher than the significance level. These results indicate that the data do not follow a normal distribution (*p* < 0.05).

Due to the non-normality of the data, nonparametric tests were performed to effectively compare the scores obtained in the questionnaire. The results of the Mann–Whitney test shown in Table 4 reveal that there is a significant difference between the total healthy lifestyle score of the students surveyed in 2020 and 2023 (*p*=0.035). It can also be observed that there is a significant difference in the score obtained in the Nutrition domain between the students of population groups M1 and M2 (*p*=0.022). No significant differences were found in the domains of Physical Activity (*p*=0.802) and Substance Consumption (*p*=0.862).

## 4. Discussion

In order to learn how the COVID-19 pandemic affected the healthy behaviors of university students, this study compared the lifestyles of medical and education students during and after the health crisis. Three key dimensions were analyzed in depth: physical activity, nutrition, and substance use. The results have allowed us to identify patterns and trends that could be relevant for the promotion of healthy habits in the university context. The results did not show significant differences in the remaining seven dimensions of the instrument. However, students showed, in general, a good level of healthy lifestyles. These findings are consistent with another study that reported a high quality of life in this population, suggesting a positive general well-being [26, 27]. Our findings are consistent with other research revealing that quarantines and social distancing protocols by COVID-19 have had a profound impact on most students as they adopted a healthier lifestyle [28, 29]. While some aspects have been negatively affected, opportunities to promote healthier habits have also been observed. The findings indicate that the combination of virtual classes and daily dietary adjustments positively influenced participants' lifestyle during the health crisis. Our results further acknowledge individual variations in the physical and emotional state of the students as a function of gender and age. Furthermore, they are in agreement with those of Pišot et al. [30], who observed healthier eating habits in the respondents.

The results of the nutrition dimension revealed that students were at a good level; however, human medicine students excelled in general. These results coincide with another study where it was concluded that students' nutrition was moderate [31]. Furthermore Espinoza-Gutierrez, Yance-Cacñahuaray, and Runzer-Colmenares [32] found that medical students during COVID-19 confinement stage opted for healthy eating habits on a regular basis and preventive behaviors, but daily physical activity was predominantly low compared to the present study whose results were good. Other studies supporting our findings indicated that good health correlates with healthy eating habits practiced [11, 22]. Consistent with our findings, the study by Perez et al. [33] emphasizes the need for students to adopt a healthy lifestyle, including physical activity or sport, adequate rest, and abstention from harmful habits. Other studies that support our findings reveal that students during COVID-19 confinements presented good to fair general health [32, 34]. In this line, we could assert that human medicine students had a better nutritional behavior, probably directed by the knowledge acquired in their professional training, since, in medicine there is a strong emphasis on wellness and health, in addition to complementing with subjects related to nutrition. Regarding physical activity, although both faculties showed good results in 2020, a decrease was observed in 2023. This reduction could be attributed to the resumption of face-to-face academic activities and the adaptation to new routines, which has led to a modification of the healthy habits acquired during confinement.

In contrast to the results obtained in the study of Health Sciences students at the Simón Bolívar University, where cardiovascular risk factors such as physical inactivity, inadequate diet, and excessive fat consumption were identified [10], our findings in human medical students show a slightly different health profile. This suggests that there are significant differences in health profiles between the two student populations. Our results indicate that students from both faculties maintained adequate dietary habits and satisfactory levels of physical activity during confinement, which is consistent with the findings of previous studies [35, 36]. Furthermore, the results suggest that students from both faculties possessed a solid foundation of knowledge and practices regarding healthy lifestyles, which enabled them to cope with confinement in a more adaptive manner. While human medical students demonstrated better performance in terms of healthy habits, this could be attributed to their academic background in health care [37]. However, data from 2023 reveal a decrease in the adoption of these healthy habits compared to previous studies [38], suggesting that, although knowledge is important, it does not guarantee consistent practice of healthy lifestyles.

Our findings show a healthy lifestyle, balanced with physical activity routines, adequate rest, and the adoption of healthy habits. This fact, together with a follow-up of health authorities' recommendations, could have played a protective role in mental health, limiting the negative influence of COVID-19. These results coincide with previous research carried out in Spain, which reported that university students had a healthy and balanced lifestyle [39]. It is important to consider that the results in university students differ from the results found in adult populations in general. Studies conducted in Argentina have shown a deterioration of healthy eating habits, which placed the population at greater risk during the pandemic [3]. Similar results were found in the United Kingdom where they found risk factors in more than 51% of the population in the pandemic stage [40], while in Mexico, sleep was affected, although they improved their diet [41]. Another study in that the same country reported improvements in physical activity but not in nutrition [42]. Other studies carried out in several countries that support our results suggest negative results that show that the increase in sedentary lifestyles affected the health of this population [43, 44].

Our findings regarding healthy lifestyles are supported by a study conducted in the capital of Peru [45], where a similar perception was observed among the population. Regarding oral hygiene habits, the results indicate that in both groups of students, they were good, which coincides with the findings of Descalzo-Casado et al. [46], who indicated that 94% of the students maintained adequate oral hygiene and cleanliness. Similarly, 46% of students in that sample stated that they did not drink alcohol or smoke, which is also related to what was found in our study. These indicators have probably improved because of the confinement, since not being able to leave the house and living with their parents allowed them to improve their lifestyles. Similarly, other studies carried out in Peru and Mexico [47, 48] indicated significant positive changes in their lifestyles, which would reaffirm what was found in our study, also indicating that the positive change was due in some way to the confinement whose restrictions played in favor of the improvement in the lifestyles of the students.

In relation to eating habits, the study of Coppi et al. [49] indicated that women showed higher intake of food in order to cope, while men preferred higher wine consumption as a coping mechanism. We observed a sharp reduction in physical activity, increased sedentary behaviors, and deterioration in sleep quality. Another study concludes that unhealthy lifestyle is prevalent among university students in whom the pandemic has produced modifications in their behaviors and lifestyles [50–53]. These results differ from those found in our research where more than 70% reported a good lifestyle. However, in 2023, students have decreased their health care, probably because they have returned to the university routine where fast food is everywhere and there is no family control over food.

## 5. Conclusion

The results of this study reveal a complex interaction between the COVID-19 pandemic and the healthy lifestyles of Peruvian university students. While medical students, thanks to their training, initially showed better eating habits and physical activity levels, the data suggest a decline in these indicators in the M2 group, highlighting the persistent challenges to maintaining healthy lifestyles in the long term, even among those with greater health knowledge. The Mann–Whitney test indicated an overall improvement in healthy lifestyle across groups, mainly attributable to improved nutritional habits during the pandemic. However, no significant changes were observed in physical activity levels or substance use. These findings highlight the need to design comprehensive interventions that not only inform about healthy habits but also address the psychosocial and environmental factors that influence the adoption of these behaviors. Furthermore, they suggest that college students, despite their youth and resilience, require ongoing support to maintain healthy lifestyles, especially in times of crisis such as a pandemic. Future research should delve deeper into the reasons behind these changes, explore the impact of different types of interventions, and assess the sustainability of the changes over the long term. The results of this study provide a solid basis for the design of health promotion programs targeting the Peruvian university population.

### 5.1. Practical Implications of the Research

Our research findings show a generally satisfactory level of healthy lifestyles among participating university students. However, the results also underline the need to strengthen and consolidate these habits in the long term. As a suggestion of practical implications, it is necessary to promote in social networks and through the university media the role of healthy eating, sports, and physical activity as determinants of well-being and health of university students. It should be specified that the implementation of these lifestyle factors reduces risk factors for cardiovascular disease and chronic diseases. Other implications of our study are that it reinforces the importance of promoting interventions by university medical center staff aimed at consolidating long-term healthy lifestyle habits and preventing depression in this student population.

### 5.2. Policies for the Promotion of Healthy Lifestyles and Habits

It is important to consider that the students have maintained their healthy habits. However, the same has not happened with their nutrition, both the students of Human Medicine and Education presented a certain neglect in their health, and this could have produced changes in their physical and mental health. Considering these results, it is necessary for the university to implement health promotion programs with the support of specialists to guide and monitor their implementation. On the other hand, the Peruvian state already has health promotion policies to promote healthy lifestyles by stages of life [52] and this should be inserted as part of the policies of the university studied. The university should adopt an active role and promote agreements with health facilities so that the student population can benefit from the programs and lectures proposed by the Peruvian Ministry of Health. To students, it is suggested that healthy daily routines include more frequent physical activity outdoors.

Public policies that seek to promote healthy lifestyles should focus on four fundamental pillars: (1) healthy eating, promoting a balanced diet that includes all nutrient groups and prioritizing the daily consumption of abundant fruits and vegetables; (2) regular practice of physical activity; (3) adequate hydration, ensuring a sufficient intake of water, which although estimated at 2 liters per day, may vary according to the level of physical activity; and (4) adequate rest, promoting a restorative sleep of 7 to 8 hours a day, in order to control stress and prevent diseases such as hypertension, diabetes, memory problems, depression and the future risk of Alzheimer's and dementia.

## Figures and Tables

**Table 1:** Distribution of the scores obtained categorized in each of the ranges for each variable of the population group surveyed during 2020.

		Healthy lifestyle score									
Variable	Group	> 40		41–58		59–69		70–80		Total	
		*n*	%	*n*	%	*n*	%	*n*	%	*n*	%
Sex	Female	5	1.35	53	14.32	85	22.97	172	46.49	315	85.1
	Male	0	0.00	10	2.70	19	5.14	26	7.03	55	14.9

Age	< 18	0	0.00	1	0.27	0	0.00	2	0.54	3	0.8
	18–24	5	1.35	59	15.95	100	27.03	186	50.27	350	94.6
	> 24	0	0.00	3	0.81	4	1.08	10	2.70	17	4.6

Faculty	Education	4	1.08	52	14.05	8	23.78	168	45.41	312	84.3
	Medicine	1	0.27	11	2.97	16	4.32	30	8.11	58	15.7

Semester	Second	1	0.27	9	2.43	5	1.35	14	3.78	29	7.8
	Sixth	1	0.27	17	4.59	35	9.46	53	14.32	106	28.6
	Tenth	3	0.81	37	10.00	64	17.30	131	35.41	235	63.5

**Table 2:** Distribution of the scores obtained categorized in each of the ranges for each variable of the population group surveyed during 2023.

		Healthy lifestyle score									
Variable	Group	> 40		41–58		59–69		70–80		Total	
		*n*	%	*n*	%	*n*	%	*n*	%	*n*	%
Sex	Female	0	0.00	10	12.20	33	40.21	20	24.39	63	76.8
	Male	0	0.00	5	6.10	7	8.54	7	8.52	19	23.2

Age	< 18	0	0.00	0	0.00	4	4.88	0	0.00	4	4.9
	18–24	0	0.00	13	15.85	28	34.15	20	24.39	61	74.4
	> 24	0	0.00	2	2.44	8	9.76	7	8.54	17	20.7

Faculty	Education	0	0.00	8	9.76	19	23.17	5	6.10	32	39.0
	Medicine	0	0.00	7	8.54	21	25.61	22	26.83	50	61.0

Semester	First	0	0.00	0	0.00	1	1.22	0	0.00	1	1.2
	Second	0	0.00	6	7.32	14	17.07	10	12.20	30	36.6
	Sixth	0	0.00	2	2.44	11	13.41	5	6.10	18	22.0
	Tenth	0	0.00	7	8.54	14	17.07	12	14.63	33	40.2

**Table 3:** Differences in students' total healthy lifestyle score (2020 and 2023), according to normality test.

Domain	Population group	Statistician	Gl	*p* value
Healthy lifestyle	M1 (2020)	0.963	370	0.001
	M2 (2023)	0.994	82	0.974

Physical activity	M1 (2020)	0.924	370	0.001
	M2 (2023)	0.942	82	0.001

Nutrition	M1 (2020)	0.934	370	0.001
	M2 (2023)	0.950	82	0.003

Substance use	M1 (2020)	0.816	370	0.001
	M2 (2023)	0.889	82	0.001

**Table 4:** Nonparametric analysis of differences in healthy lifestyle domains in college students before and after the COVID-19 pandemic.

Domain	Population group	Mean	Average rank	Sum of ranks
Healthy lifestyle	M1 (2020)	67.71	232.61	86,066.00
	M2 (2023)	65.95	198.93	16,312.00
Physical activity	M1 (2020)	3.11	225.79	83,543.00
	M2 (2023)	3.17	229.70	18,835.00
Nutrition	M1 (2020)	5.45	233.04	86,223.50
	M2 (2023)	5.01	197.01	16,154.50
Substance use	M1 (2020)	8.18	226.01	83,624.00
	M2 (2023)	8.45	228.71	18,754.00

	***U***	**W**	***Z***	**p**

Healthy lifestyle	12,909.00	16,312.00	−2.114	0.035
Physical activity	14,908.00	83,543.00	−0.251	0.802
Nutrition	12,751.50	16,154.50	−2.293	0.022
Substance use	14,989.00	83,624.00	−0.174	0.862

*Note:U* = Mann–Whitney test; *W* = Wilcoxon test.

## Data Availability

Data supporting the results of this study are available from the corresponding author upon request.
